# Malignant Pheochromocytoma Presenting as a Large Adrenal Mass With Cavoatrial Tumor Thrombus

**DOI:** 10.1016/j.aace.2024.02.001

**Published:** 2024-02-08

**Authors:** Run Yu, Martin S. Allen-Auerbach, Michael W. Yeh

**Affiliations:** 1 Division of Endocrinology, UCLA David Geffen School of Medicine, Los Angeles, California; 2 Ahmanson Translational Imaging Division, Department of Molecular and Medical Pharmacology, UCLA Medical Center, UCLA David Geffen School of Medicine, Los Angeles, California; 3 Section of Endocrine Surgery, UCLA David Geffen School of Medicine, Los Angeles, California

### Case Presentation

A 50-year-old female with newly-diagnosed bilateral breast cancer presented with a large right adrenal mass and tumor thrombus in the inferior vena cava extending into the right atrium. A few days before presentation, abdominal ultrasound for evaluating abdominal pain had found a 9-cm right adrenal mass. Breast cancer metastasis was suspected. Fluorodeoxyglucose-positron emission tomography/computed tomography (FDG-PET/CT) confirmed the large right adrenal mass which exhibited intense FDG uptake with maximal standard uptake value (SUVmax) of 33.6 and high noncontrast Hounsfield units (>40) (Fig. 1, red arrows). Furthermore, FDG-PET/CT revealed a tumor thrombus in the inferior vena cava extending into the right atrium (Fig. 1, yellow and blue arrows). A left breast lesion was also noted with SUVmax of 5 (Fig. 1, green arrow). No other FDG-avid lesions were found. Magnetic resonance imaging demonstrated a heterogeneously enhancing right adrenal mass with intra-tumoral cystic degeneration and a contiguous tumor thrombus growing into the right atrium through the inferior vena cava (Fig. 1). The patient denied history of hypertension but recalled recent episodes of "panic attack" characterized by palpitations and feelings of dread. She also complained of recent fatigue, abdominal pain, and weight loss of 10 lbs. She denied hirsutism. Her family history was positive for breast cancer and colon cancer but negative for endocrine tumors. Physical examination results were unremarkable. Plasma normetanephrine was 10.71 nmol/L (normal <0.89) and metanephrine <0.1 nmol/L (<0.49).

**Fig. 1:**
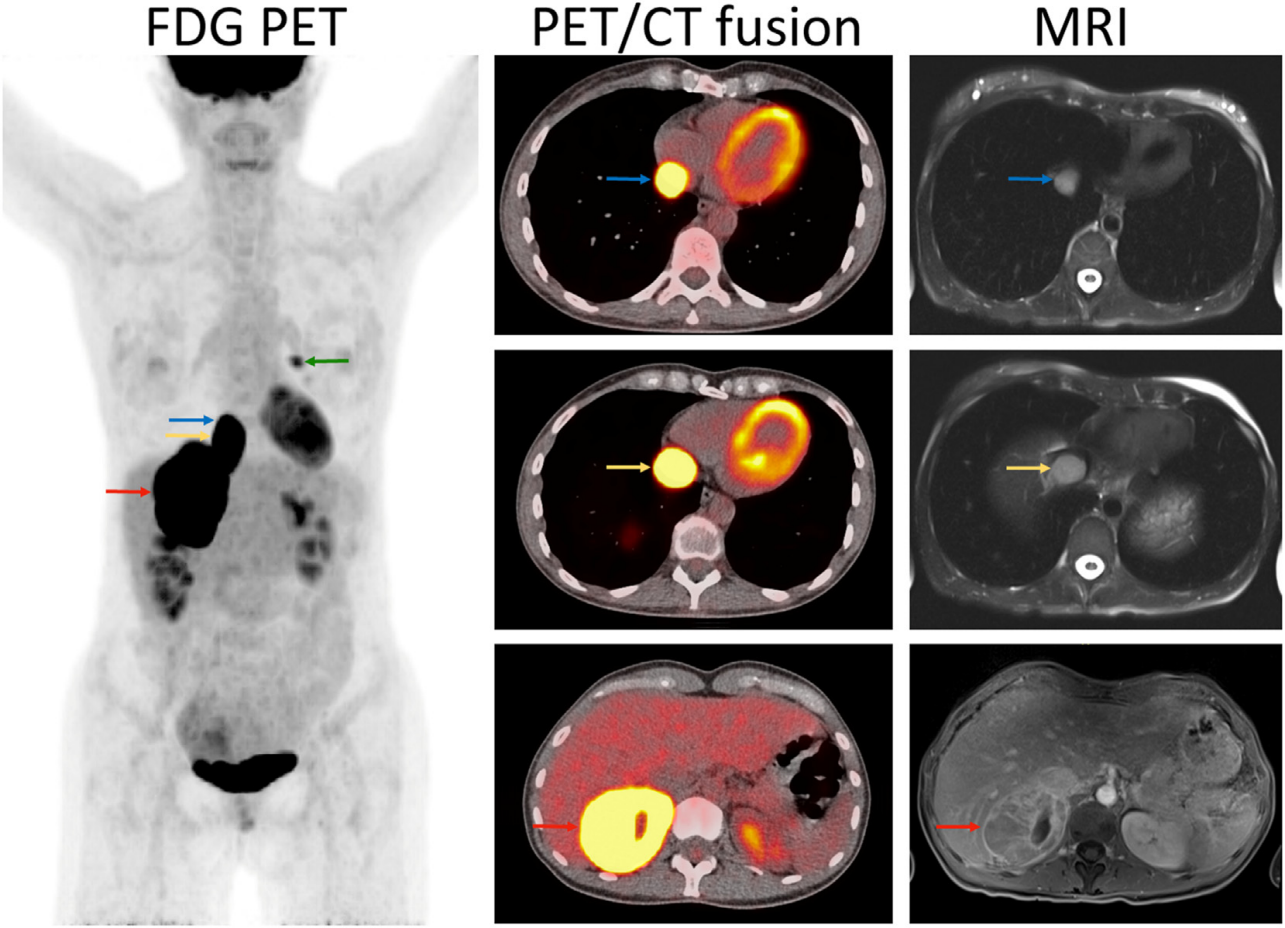
Imaging findings. See text for detail.

### What is the Diagnosis?

#### Answer

Malignant pheochromocytoma with cavoatrial tumor thrombus. Pheochromocytoma is frequently encountered in patients with suspected adrenal metastasis but there are no clear clinical predictors of pheochromocytoma in such patients.^1^ Consideration of pheochromocytoma in the differential diagnosis is thus key to the correct diagnosis of adrenal mass. Malignant pheochromocytoma characterized by tumor thrombus is very rare and poses surgical challenges due to vascular involvement.^2^^,^^3^ After alpha and beta blockage, the patient underwent open en bloc resection of the pheochromocytoma (9.2 cm) and tumor thrombus under cardiopulmonary bypass with circulatory arrest (Fig. 2). Histological examination confirmed pheochromocytoma with high PASS (Pheochromocytoma of the Adrenal gland Scaled Score) of 12 (a score≥4 is consistent with malignant behavior). The patient recovered well postoperatively and underwent bilateral mastectomy a few months later. Genetic test of a panel of 88 genes associated with hereditary tumors showed heterozygous variants of unclear significance in *FH* (a pheochromocytoma susceptibility gene) and *RECQL* (a breast cancer susceptibility gene). She had no evidence of recurrence or metastasis 1 year after the pheochromocytoma resection. This case highlights that pheochromocytoma and even malignant pheochromocytoma can be encountered in patients with suspected adrenal metastasis and biochemical tests for pheochromocytoma should be performed in those patients.

**Fig. 2:**
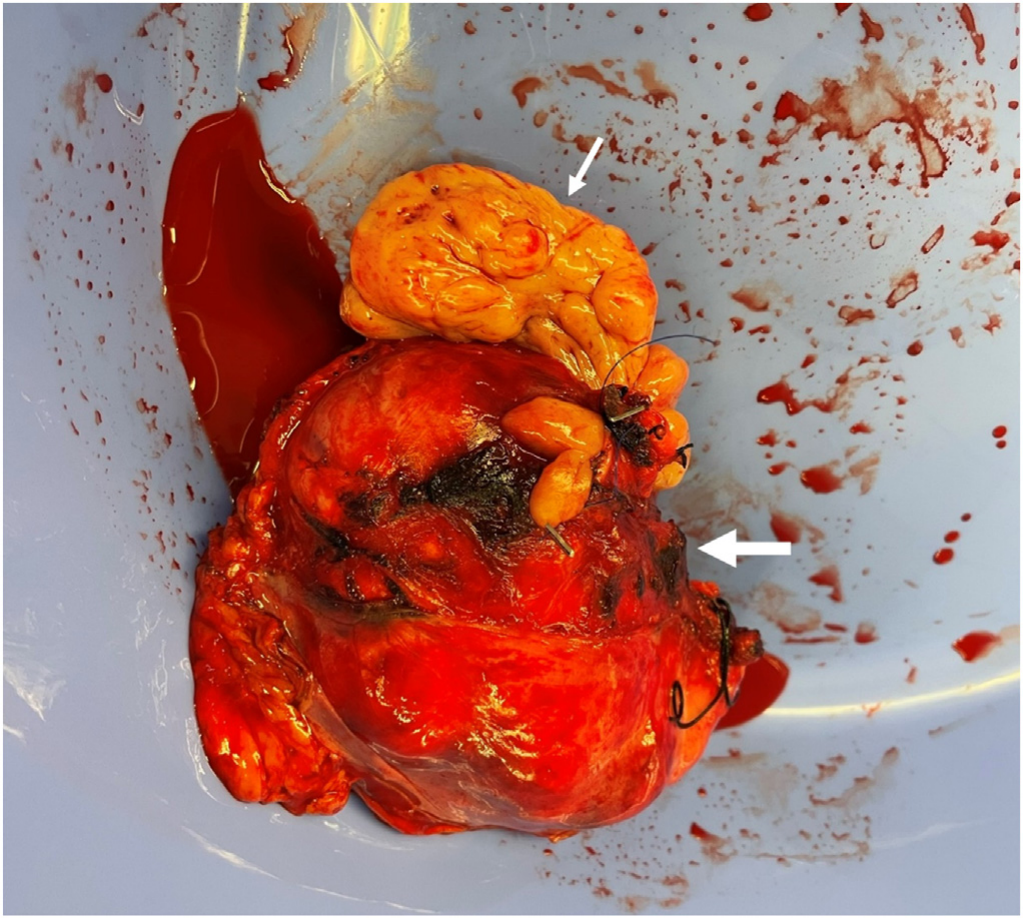
Resected specimen. Wide arrow: right adrenal pheochromocytoma; thin arrow: tumor thrombus.

## Disclosure

The authors have no multiplicity of interest to disclose.
